# Factors affecting the infant antibody response to measles immunisation in Entebbe-Uganda

**DOI:** 10.1186/1471-2458-13-619

**Published:** 2013-07-01

**Authors:** Dennison Kizito, Robert Tweyongyere, Alice Namatovu, Emily L Webb, Lawrence Muhangi, Swaib A Lule, Henry Bukenya, Stephen Cose, Alison M Elliott

**Affiliations:** 1 Co-infection Studies Programme, MRC/UVRI Uganda Research Unit on AIDS, Uganda Virus Research Institute, P.O. BOX 49, Entebbe, Uganda; 2 College of Veterinary Medicine, Animal Resources and Biosecurity, Makerere University, P.O. BOX 7062, Kampala, Uganda; 3 London School of Hygiene and Tropical Medicine, Keppel Street, London WC1E 7HT, UK; 4 Expanded Programme on Immunisation Laboratory, Uganda Virus Research Institute, P.O. BOX 49, Entebbe, Uganda

**Keywords:** Infections, Co-infections, Measles, Helminth, Malaria, HIV, Maternal, Infants, Pregnancy, Immunisation

## Abstract

**Background:**

Vaccine failure is an important concern in the tropics with many contributing elements. Among them, it has been suggested that exposure to natural infections might contribute to vaccine failure and recurrent disease outbreaks. We tested this hypothesis by examining the influence of co-infections on maternal and infant measles-specific IgG levels.

**Methods:**

We conducted an observational analysis using samples and data that had been collected during a larger randomised controlled trial, the Entebbe Mother and Baby Study (ISRCTN32849447). For the present study, 711 pregnant women and their offspring were considered. Helminth infections including hookworm, *Schistosoma mansoni* and *Mansonella perstans,* along with HIV, malaria, and other potential confounding factors were determined in mothers during pregnancy and in their infants at age one year. Infants received their measles immunisation at age nine months. Levels of total IgG against measles were measured in mothers during pregnancy and at delivery, as well as in cord blood and from infants at age one year.

**Results:**

Among the 711 pregnant women studied, 66% had at least one helminth infection at enrolment, 41% had hookworm, 20% *M. perstans* and 19% *S. mansoni*. Asymptomatic malaria and HIV prevalence was 8% and 10% respectively. At enrolment, 96% of the women had measles-specific IgG levels considered protective (median 4274 mIU/ml (IQR 1784, 7767)). IgG levels in cord blood were positively correlated to maternal measles-specific IgG levels at delivery (r = 0.81, p < 0.0001). Among the infants at one year of age, median measles-specific IgG levels were markedly lower than in maternal and cord blood (median 370 mIU/ml (IQR 198, 656) p < 0.0001). In addition, only 75% of the infants had measles-specific IgG levels considered to be protective. In a multivariate regression analysis, factors associated with reduced measles-specific antibody levels in infancy were maternal malaria infection, infant malaria parasitaemia, infant HIV and infant wasting. There was no association with maternal helminth infection.

**Conclusion:**

Malaria and HIV infection in mothers during pregnancy, and in their infants, along with infant malnutrition, may result in reduction of the antibody response to measles immunisation in infancy. This re-emphasises the importance of malaria and HIV control, and support for infant nutrition, as these interventions may have benefits for vaccine efficacy in tropical settings.

## Background

Despite the availability of a stable and effective vaccine, measles outbreaks are still an important concern in Eastern and Southern Africa. Although the Expanded Programme on Immunisation (EPI) operates in many countries, there are still issues that affect complete coverage in many countries, and this also contributes to the continuing measles outbreaks throughout the world [1,2]. To prevent serious outbreaks and deaths in the developing world, measles vaccinations are administered at 9 months of age [3]. Previous studies have suggested that induction of protective immunity against measles by immunisation in infants may be influenced by a number of factors, including the rate of decay of maternally acquired measles-specific antibodies [4], and maternal infection with other pathogens during pregnancy [5-9]. Age at immunisation [10,11], number of doses and the measles vaccine strain used [12,13] can also greatly influence the levels of antibody response. In addition, it has been hypothesised that helminth infections might impact on the immunogenicity and efficacy of vaccines [14] and the ability of the host to respond to infections with other pathogens [15]. Moreover, it has been hypothesised that *in utero* exposure of the child to maternal helminth infection may have a long-term effect on the child’s immunological development, including their response to immunisation [16,17]. Contrary to this hypothesis, within the Entebbe Mother and Baby study (EMaBS), we have shown that anthelminthic treatment during pregnancy had no effect on infant antibody levels following measles immunisation [18]. However, we considered the possibility that maternal helminths might have other effects on the infant response that are not modified by treatment during pregnancy, or that other chronic immunomodulating infections such as HIV or malaria may influence the infant response to immunisation. We therefore explored these possibilities in an observational analysis within the EMaBS cohort, which had been established for a trial of anthelminthic treatment during pregnancy in Entebbe, Uganda.

## Methods

### Study setting and design

The Entebbe Mother and Baby Study (EMaBS) was a larger, randomised, double-blind placebo controlled trial of treatment of helminths in pregnancy with albendazole versus placebo and praziquantel versus placebo in a 2x2 factorial design involving 2507 pregnant women and their infants (trial registration number ISRCTN32849447). The study design and trial results have previously been reported [18,19].

For this study we conducted an observational analysis using samples and data that had been collected during the EMaBS. The aims of this observational analysis were

1. to investigate the hypothesis that maternal helminth infections influence maternal anti-measles antibody levels, and the infant response to measles immunisation, and

2. to investigate other factors associated with the infant response to measles immunisation

Briefly, the study was based in Entebbe Hospital and recruited participants from Entebbe municipality and the adjacent Katabi sub-county, a population comprising urban, rural and fishing communities. Pregnant women in the second or third trimester were enrolled at Entebbe Hospital antenatal clinic if they were resident in the study area, planning to deliver in the hospital, willing to know their HIV status and willing to take part in the study. They were excluded if they had evidence of possible helminth-induced pathology (severe anaemia, clinically apparent liver disease, bloody diarrhoea), if the pregnancy was abnormal, or if they had already enrolled during a previous pregnancy [18]. Women gave written informed consent for their own participation and for the participation of their infant in the study.

Women were followed up at delivery. Babies were followed up at immunisation visits, at age six, nine and 12 months and quarterly thereafter to age five years; annual follow up is still on-going.

The babies were immunised at birth with the Bacille Calmette Guerin (BCG) and oral polio (OPV) vaccines; at 6, 10 and 14 weeks with OPV, *Diphtheria*, tetanus, *Pertussis*, *Haemophilus influenzae*, hepatitis B vaccines; and at nine months with measles vaccination. Immunisations were usually given at the hospital immediately after delivery (BCG and OPV), or at the hospital outpatient department adjacent to the research follow-up clinic. The place at which the immunisation was given (Entebbe Hospital or elsewhere) was documented. During the study period measles vaccines used at Entebbe Hospital were Edmonston Zagreb strain (Serum Institute India Ltd), Edmonston Zagreb strain (Measles Vaccine Vaksin) from Biofarma, Indonesia and Schwarz strain (Rouvax) from Sanofi Pasteur. The strain and manufacturer of vaccines given to individual infants was not recorded.

### Samples and laboratory methods

#### Sample collection

Samples collected were from mothers during pregnancy and at delivery (stool and blood), from cord blood, and from infants at age one year (stool and blood). Stool and blood samples were used for diagnosis of intestinal and systemic helminth infections, and blood samples for malaria slides and HIV serology. Serum was aliquotted and stored frozen (^-^80°C) at Uganda Virus Research Institute (UVRI) until the time of the measles antibody assay. In addition, samples were collected from HIV-exposed infants at age 6 weeks and 18 months, for HIV-specific PCR and serology, respectively. Infants were not sampled at immunisation, so the effect of maternal anti-measles antibody or of malaria or HIV infection at the time of immunisation on the induction of immune response to the vaccine could not be determined.

#### Sample selection for measles analysis

The current observational analysis involved measurement of measles-specific antibody levels for 711 mother-baby pairs who had blood samples available from each of four time points: from the mother at enrolment into the study during the second or third trimester of the pregnancy, from the mother at delivery, from cord blood, and from the infant at age one year. Mother-baby pairs were included if the maternal delivery samples were obtained within seven days after delivery and if the baby had a clearly documented record of measles immunisation at 9 months, administered in Entebbe Hospital. One from each set of twins was excluded from the analysis.

#### Measurement of measles antibody

Levels of measles-specific IgG antibody in serum was measured by ELISA using a quantitative commercial kit (Enzygnost, Germany) according to the manufacturer’s protocol. Briefly, serum samples were added in duplicate to a microtitre plate, which contained two parallel wells coated with either whole measles virus antigen, or control antigen derived from non-infected cells. The testing kit had an “anti-measles virus-reference” (Human serum containing IgG antibodies to measles virus antigens) that was included on each test run. The test kit had a sensitivity of 99.6% and specificity of 100%, and could accurately test samples containing a minimum 150 mIU/ml.

The serum samples were tested in a randomised sequence, but with all samples from each mother-baby pair on the same plate. Measles-specific IgG antibody levels were quantified using the α-method, reported in milli international units per millilitre (mIU/ml) of serum or plasma, by the following formula: log_10_ mIU/ml = α x A^β^ (where α and β are lot-dependent constants, provided by the manufacturer [6]). The values thus calculated reflected the international standard for anti-measles serum (1st international standard) of the WHO. A protective response was defined as having a level of measles antibody greater than 200 mIU/ml as reported elsewhere [4,18,20].

#### Parasitological procedures

Stool samples were collected before the study drug was given to the study participants at the antenatal clinic in Entebbe Hospital and were examined using the Kato Katz method for helminth ova including hookworm and *S. mansoni*[21,22]*.* The charcoal culture method was used to examine for *Strongyloides stercoralis*[23-26]*.* Whole blood samples were examined for *M. perstans* according to a modified Knott’s method [27] and for malaria by Leishman stained thick smears.

#### HIV screening

HIV sero-status of the women was determined at enrolment into the study using a serial rapid testing algorithm as previously reported [25]. For offspring of HIV positive women, HIV viral load was measured at six weeks of age using both DNA PCR and quantitative RT-PCR, to determine vertical HIV transmission. HIV antibody testing in infants was done at 18 months. Children were defined as HIV infected if found positive on both PCR assays at 6 weeks, or on serology at 18 months, and as exposed uninfected if found negative [18,28].

#### Data analysis

Data were analysed using Stata version 10 (College Station, Texas,USA) with the following objectives: (1) to compare measles antibody levels at the four time points (maternal blood during pregnancy and after delivery, cord blood, and at age one year); (2) to determine the associations between maternal socio-demographic characteristics, infections (helminths, HIV, malaria) and maternal antibody levels; (3) to determine the associations between maternal and childhood characteristics, helminth infections, HIV, malaria and the infant response to measles immunisation.

Characteristics of the study population were summarised using frequencies for categorical variables, with means and medians for continuous variables. Antibody levels showed skewed distributions and were therefore log-transformed for analysis. Correlations between log antibody levels at each time point were examined using Pearson’s correlation coefficient. Paired t-tests were also done to assess whether the actual levels of antibody differed between each time point. Chi-squared tests were used to examine associations between maternal/child characteristics and presence of protective antibody levels. Simple linear regression was then used to examine crude associations between each potential risk factor and maternal log antibody levels, and between each potential risk factor and infant log antibody levels. The following variables were examined for possible association with maternal measles antibody levels: maternal age, education, marital status, maternal tribe, socio-economic status, gravidity, HIV status, CD4 counts, asymptomatic malaria, worm infection, worm infection intensity, and gestation stage at enrolment. The same variables were considered for possible association with infants’ measles-specific antibody levels, with the addition of baby’s birth weight, baby’s sex, number of malaria episodes during infancy, infant asymptomatic malaria at age one year, infant HIV, and wasting and stunting at one year of age. Multivariable linear regression models were then developed for each factor that showed an association with antibody levels with a p-value <0.1 in crude analysis. Using a causal diagram, these models were adjusted for any distal or concurrent variables that also showed some evidence of association with the outcome based on crude analysis. For example, associations between maternal infections and maternal antibody levels were adjusted for any maternal socio-demographic factors or concurrent infections that showed association with antibody levels in the crude analysis, but associations between socio-demographic factors and maternal antibody levels were not adjusted for infections since these may be on the causal pathway between the socio-demographic factor and antibody levels.

#### Ethical considerations

Ethical approval was granted by the Uganda Virus Research Institute Science and Ethics Committee, the Ethics committee of the London School of Hygiene and Tropical Medicine, and the Uganda National Council of Science and Technology.

## Results

Among the 711 mothers included in this study, 41% had hookworm at enrolment, 20% *M. perstans*, and 19% *S. mansoni.* Sixty six percent of the women had at least one helminth species. Among women with hookworm and *S. mansoni,* 4% and 10% respectively had heavy infection intensities. The prevalence of maternal asymptomatic malaria and maternal HIV was 8% and 10% respectively. Of the 74 HIV positive mothers, 22 had CD_4_ counts less than 350 cells/μl. The mean age of women at enrolment was 24 years with the youngest being 14 years and the oldest 47 years. Median maternal gestational age was 26 weeks at enrolment. Half of the pregnant women had attained at least a primary level education. Almost 58% of the women reported having had more than two previous pregnancies. At least 98% of the women had a full term delivery (38–42 weeks gestation). The mean number of people per household was 3.7 and the mean household income was less than 30,000 Uganda Shillings per month.

Mothers who were included in this study were on average older and more educated, and were less likely to be infected with hookworm and malaria, than those EMaBS cohort members who were not included in this study. There were no other significant differences between the two groups.

The mean birth weight of children in this sample was 3.2 kg (range of 1.3-4.9 kg). At age one year, 92 (13%) children were considered to be stunted and 27 (4%) had evidence of wasting. No cases of underweight infants were observed. Sixty-two children (9%) were exposed to HIV but were uninfected and 12 (2%) were HIV infected. Forty children (6%) at age one year had asymptomatic malaria infection.

### Maternal and cord measles antibody levels

**Figure 1:**
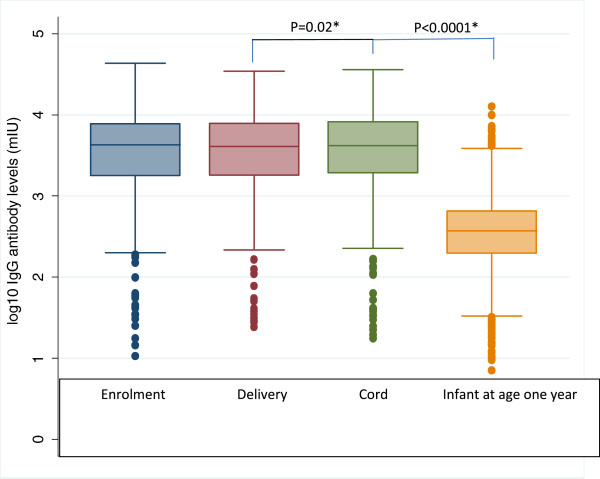
**Measles-specific IgG antibody levels in cord, infants and their mothers.** Paired t-test values.

The Median (IQR) measles-specific antibody levels in mothers at enrolment and delivery, and in cord blood, were very similar (4274 mIU /ml (IQR 1784, 7767), 4079 mIU/ml (IQR 1802, 7854) and 4176 mIU/ml (IQR 1932, 8247), respectively), although the mean level at delivery was slightly lower than the level in cord blood (paired t-test p = 0.02; Figure 1). Maternal measles-specific IgG antibody levels were highly correlated between enrolment and delivery (r = 0.81; p < 0.0001). Maternal and cord blood measles specific IgG levels were also highly correlated (r = 0.82; p < 0.0001). Of the women enrolled in the study, 96% had measles-specific IgG levels within the protective range at enrolment.

In crude linear regression analyses of the factors associated with maternal measles antibody levels at enrolment in this cohort, we found that only maternal age, gravidity, and asymptomatic malaria infection were associated with antibody levels (p < 0.1; Table 1). However, in multivariable models, the association with gravidity was no longer statistically significant after adjusting for maternal age, and the association with malaria was no longer statistically significant after adjusting for maternal age and gravidity. Thus, only maternal age at the time of enrolment in the study was associated with increased measles-specific IgG levels (Table 1).

**Table 1:** Factors associated with measles antibody levels in mothers during pregnancy

**Univariable analyses**					**Multivariable analyses**^**1**^	
**Explanatory variable**	**Status**	**Mean(SD) log IgG**	**Mean difference (95% confidence interval)**	**P-value**	**Mean difference (95% confidence interval)**	**P-value**^**2**^
Gravidity	1	3.42 (0.55)		0.002		0.62
	2-4	3.54 (0.49)	0.13 (0.04, 0.22)		0.05 (−0.05, 0.15)	
	5+	3.61 (0.47)	0.20 (0.08, 0.31)		0.06 (−0.10, 0.22)	
Asymptomatic malaria	No	3.53 (0.50)		0.07		0.26
	Yes	3.40 (0.59)	−0.13 (−0.27, 0.01)		−0.08(−0.22, 0.06)	
Maternal age in years	14-19	3.38 (0.52)		0.0005		0.0005
	20-24	3.52 (0.51)	0.14 (0.04, 0.24)		0.14 (0.04, 0.24)	
	25-29	3.57 (0.49)	0.19 (0.08, 0.30)		0.18 (0.08, 0.29)	
	30-34	3.65 (0.44)	0.27 (0.14, 0.40)		0.27 (0.14, 0.40)	
	35+	3.57 (0.46)	0.19 (−0.01, 0.39)		0.18 (−0.01, 0.38)	

*SD* standard deviations; ^1^in multivariable analyses, asymptomatic malaria was adjusted for age and gravidity, gravidity was adjusted for age, age was unadjusted ^2^likelihood ratio test; the mean IgG levels observed at maternal enrolment were similar to maternal delivery and cord blood levels.

Of note, maternal HIV, maternal helminth infections and anthelmintic treatment during pregnancy showed no associations with maternal or cord blood measles antibody levels (data not shown). Similar trends were observed for measles antibody levels in maternal blood at delivery and in cord blood.

### Measles antibody levels at age one year

Measles-specific IgG antibody levels among the infants at age one year were significantly lower than maternal or cord blood levels (median 370 mIU/ml (IQR 198, 656), paired t-test on logged values p < 0.0001; Figure 1). They showed no correlation with maternal (r = −0.05; p = 0.14) or cord blood levels (r = −0.02; p = 0.54). Of particular concern, only 75% of the infants at age one year were found to have antibody levels considered protective against measles.

In a multivariable analysis of factors associated with the infant response to measles immunisation, we found that higher maternal gravidity was associated with higher measles-specific IgG levels, while maternal asymptomatic malaria was associated with lower levels in the offspring (Table 2). Infant wasting showed a negative association with measles-specific IgG levels and infants infected with malaria or HIV also had lower antibody levels than their uninfected counterparts. Maternal helminth infections showed no associations with infant measles-specific IgG levels. As reported elsewhere [19], levels of total measles-specific IgG at age one year was not affected by maternal anthelminthic treatment.

**Table 2:** **Multivariable analysis of factors associated with infants measles log**_**10**_** antibody responses at one year of age, following immunisation at 9 months**

	**Univariable analysis**						**Multivariable analysis**^**1**^	
**Explanatory variable**	**Status**	**Proportion (%) with protective antibody levels**	**P-value**^**2**^	**Mean (SD) Log**_**10**_**antibody levels**	**Difference (95% confidence interval)**	**P-value**	**Difference (95% confidence interval)**	**P-value**^**3**^
Gravidity	1	147/184 (80)	0.009	2.54 (0.48)		0.06		0.06
	2–4	310/413 (75)		2.55 (0.51)	0.01 (−0.08, 0.10)		0.01 (−0.08, 0.10)	
	5+	73/114 (64)		2.42 (0.57)	−0.12 (−0.24, 0.001)		−0.12(−0.24, 0.001)	
Maternal malaria infection	No	484/646 (75)	0.3	2.54 (0.51)		0.09		0.05
	Yes	37/54 (68)		2.41 (0.67)	−0.13 (−0.27, 0.02)		−0.14 (−0.29, 0.002)	
Infant asymptomatic malaria at age one year	No	486/647 (75)	0.07	2.54 (0.51)		0.003		0.003
	Yes	25/40 (62)		2.29 (0.59)	−0.25 (−0.42, -0.09)		−0.25 (−0.41, -0.08)	
Infant HIV	Unexposed	482/637 (76)	0.003	2.54 (0.50)		0.002		0.003
	Exposed, uninfected	44/62 (71)		2.48 (0.54)	−0.06 (−0.19, 0.07)		−0.05 (−0.18, 0.09)	
	Infected	4/12 (33)		2.02 (0.85)	−0.52( −0.82, -0.23)		−0.50 (−0.80, -0.21)	
Weight-for length/height (wasting)	No	514/684 (75)	0.06	2.54 (0.50)		0.01		0.04
	Yes	16/27 (59)		2.28 (0.78)	−0.26 (−0.45, -0.06)		−0.21 (−0.41, -0.01)	

*SD* Standard deviation; ^1^in multivariable analyses, maternal asymptomatic malaria was adjusted for gravidity, infant asymptomatic malaria and HIV were adjusted for each other and for gravidity and maternal malaria infection, wasting was adjusted for all other variables in the table ^2^chi2 test ^3^likelihood ratio test.

## Discussion

Measles-specific IgG levels are widely used as proxy measures of immunity in measles immunisation programmes. The presence of measles-specific IgG antibodies in sera indicates either a previous infection, or successful immunisation [1,20,29,30]. This current study presents an observational analysis of the associations between co-infections and levels of measles-specific IgG antibodies in a cohort of pregnant women and their infants. Our findings indicate that maternal helminth infection during pregnancy does not influence the levels of measles-specific antibodies in their offspring at age one year. Previous studies have suggested that maternal helminth infections might alter the immunological balance between Th1, Th2 and Treg pathways by preventing Th1 responses and promoting a Th2 bias, thereby impairing responses to viral vaccines given to infants [15,31]. However, neither this observational analysis, nor our other published data [18], support the argument of Labeaud *et al.* that treatment of antenatal helminth infections would improve infant responses to immunisations [14].

We found that infants born of mothers infected with malaria had reduced levels of measles-specific IgG at age one year when compared with infants of uninfected mothers. Malaria is well known to be immunosuppressive [32] and it may be that malaria infection in the mother creates an intra-uterine environment that has a long-term effect on the infant response to infection and immunisation. However, in this cohort, as in other studies [33], maternal malaria was also associated with infant malaria incidence (data not shown), so this result might also simply reflect a direct effect of higher infant exposure to malaria. In keeping with this hypothesis, infants with asymptomatic malaria also had lower anti-measles antibody levels than uninfected infants. Our findings are in apparent contradiction to a previous study performed in Guinea Bissau in 1983/4 in children aged 8–19 months that showed an increase in measles-specific responses after a malaria infection [34]. However, in this paper, the authors used measles infected Vero cells as the target antigen for their ELISA, whereas we used a commercial kit that supplies purified measles protein. It is possible that both studies are correct, as presumably the study by Smedman and colleagues may have more measles epitopes to bind antibody than that supplied in the standard commercial kits of today. Moreover, the timing of assessments was different between the two studies, with most children in the earlier study infected with malaria at the time of measles immunisation. In our study the malaria assessment was made at the time of assessing the antibody response. We do not know whether our infants had malaria at the time of immunisation.

With regard to maternal HIV infection, measles-specific antibody levels were similar in infants of uninfected women and in HIV-exposed but uninfected infants, but HIV infected infants had much lower antibody levels. The infants’ HIV status was defined based on samples obtained at 6 weeks and/or 18 months of age – not at nine months when immunisation was given, nor at one year when the measles antibody assays were performed. This means that a small number of infants may have been misclassified; if this is so then our result, although striking, may have underestimated the adverse effect of HIV positivity in the child. Although our study included only a small number of HIV infected infants, these findings are consistent with studies performed elsewhere [8,9,35,36]. However, our findings for measles immunisation in HIV exposed but uninfected children were different from those reported for hepatitis B, diphtheria and tetanus immunisation in a study in Brazil in which antibody responses were found to be reduced in this group [37]. This may be related to differences in effect between live and subunit vaccines: within EMaBS we also found that HIV exposed, uninfected infants had reduced cellular responses to tetanus toxoid following tetanus immunisation, but not to mycobacterial antigen following BCG immunisation [38].

Infant wasting (low weight for length) was associated with reduced measles-specific IgG levels. Infant wasting could be attributable to poor nutrition, which clearly has detrimental effects on the immune system, and this may explain why we saw an effect of infant wasting. This, again, is in apparent contradiction with an earlier study [39], but may again be reconciled by the use of different techniques to calculate measles titres. In their study, McMurray and colleagues used a common technique at the time to measure measles antibody titres, the hemagglutanin inhibition (HI) assay. At around the same time, Nuemann and colleagues [40] published comparison data showing that both antihemolysin (AH) and ELISA techniques were more sensitive and specific than the traditional HI assay. It is thus possible that the original HI assay may be less sensitive and specific than the kits of today, leading the authors to conclude that malnutrition had no effect on measles antibody titres in comparison to our data.

Our study therefore suggests that malaria, HIV and nutrition have more important implications for levels of measles-specific antibodies in the first year of life than helminth infections. Indeed, in places of high HIV prevalence and incidence, HIV infected infants are suspected to contribute to the transmission and sustainability of measles virus outbreaks in the sub Saharan region [1].

In Uganda, the national measles immunisation coverage (routine and campaign) increased from 63% in 2001 to 90% in 2004, and a slight drop to 85% in 2007 [41]. The current coverage is estimated to be at 71%, although the national sero-conversion rates among immunised children is not known. We found that measles-specific antibody levels in the infants were much lower than the levels in their mothers. This is presumed to be due to the fact that infants had not been exposed to natural measles infection. In this study period (2003 to 2005) there were only two cases of measles confirmed amongst the cohort before the nine month vaccination [18], therefore actual cases are unlikely to have had an impact on our results, and there was no reported measles outbreak in the country [42]. In addition, none of the infants included in this study had prior documented measles immunisation below 9 months of age. All infants who presented with a measles-like illness were tested for antibodies and of these, a small number had rubella IgG antibodies and the rest were not infected with either rubella or measles. However, despite the fact that all infants who participated in this study were immunised at nine months of age at Entebbe district referral hospital with proper monitoring, only 75% of the infants at one year of age had measles-specific IgG levels considered protective against measles infection. The reasons for this poor response are not clear. One possible explanation might lie in the high levels of maternal antibody, which is well known to interfere with infant responses to measles immunisation. Although we did not collect information on maternal measles immunisation, the high titres in the mothers might imply that they had multiple exposures to the measles virus, either through immunisation and exposure to the natural infection, or multiple exposures to the natural infection. Such a scenario may easily boost the immune response to measles and maintain antibody titres at a high level.

Other possible explanations include inherent (for example genetic) differences among the immunised infants (which we are currently exploring) or differences in the strain of vaccine itself, as well as the potency of the vaccine at the time of administration. These issues surrounding the vaccine strain or potency we were unable to exclude, as this information was not captured at the time of immunisation, and the hospital itself does not keep records of such information.

## Conclusions

In conclusion, we have found no evidence that maternal helminth infection influences the infant response to measles immunisation, whereas control of malaria and HIV infection in mothers and their infants, and support for infant nutrition, may have benefits for measles vaccine efficacy. The high proportion of infants in whom the vaccine failed to induce a protective response (25%) supports the need for measures aimed at improving the efficacy of initial measles immunisation, and for revaccination campaigns, in Sub-Saharan Africa.

## Competing interests

All authors declare that they have no competing interests.

## Authors’ contributions

DK led the manuscript draft writing and incorporating the co-author’s comments. DK, RT, SC, ELW, & AME designed the study, analysis and writing of the manuscript. DK, RT, AN, & HB participated in sample processing, carried out the immunoassays and analysis. ELW & LM performed the statistical analysis. SAL was responsible for overseeing the clinical procedures. All authors have read and approved the manuscript.

## Authors’ information

DK MSc, Scientific officer, MRC/UVRI Co-infections Studies programme, RT PhD, Principal Investigator and Senior Lecturer, College of Veterinary medicine, Animal resources and Biosecurity, Makerere University, AN MSc, Lecturer, College of Veterinary medicine, Animal resources and Biosecurity, Makerere University, ELW PhD, Trial Statistician and Lecturer, London School of Hygiene and Tropical Medicine, LM MA(Econ), Trial statistician and data base manager, MRC/UVRI Co-infections Studies programme, SAL MBChB, study clinician, MRC/UVRI Co-infections Studies programme, HB BSc, Technical Laboratory Supervisor, Expanded Programme on Immunisation Laboratory, Uganda Virus Research Institute, SC PhD, Senior Immunologist, MRC/UVRI Co-infections Studies programme and Lecturer in Immunology, London School of Hygiene and Tropical Medicine, and AME MD, Head MRC/UVRI Co-infections Studies programme, and Professor of Tropical Medicine, London School of Hygiene and Tropical Medicine*.*

## Pre-publication history

The pre-publication history for this paper can be accessed here:

http://www.biomedcentral.com/1471-2458/13/619/prepub

## References

[B1] Moss WJ Griffin DE **Measles** Lancet 2011 10.1016/S0140-6736(10)62352-5

[B2] WHO-UNICEF Joint WHO and UNICEF press release measles outbreaks in Eastern and Southern Africa Wkly Epidemiol Rec 2010 Geneva: WHO/UNICEF

[B3] WHO WHO AFRO Measles SIA Field Guide World Health Organisation 2006 Geneva: WHO

[B4] Naniche D Human immunology of measles virus infection Curr Top Microbiol Immunol 2009 330 151 171 10.1007/978-3-540-70617-5_8 19203109

[B5] de Moraes-Pinto MI Verhoeff F Chimsuku L Milligan PJ Wesumperuma L Broadhead RL Brabin BJ Johnson PM Hart CA Placental antibody transfer: influence of maternal HIV infection and placental malaria Arch Dis Child Fetal Neonatal Ed 1998 79 3 F202 205 10.1136/fn.79.3.F202 10194992 PMC1720856

[B6] Dopatka HD Giesendorf B Single point quantification of antibody by ELISA without need of a reference curve J Clin Lab Anal 1992 6 6 417 422 10.1002/jcla.1860060614 1432369

[B7] Okoko BJ Wesuperuma LH Ota MO Banya WA Pinder M Gomez FS Osinusi K Hart AC Influence of placental malaria infection and maternal hypergammaglobulinaemia on materno-foetal transfer of measles and tetanus antibodies in a rural west African population J Health Popul Nutr 2001 19 2 59 65 11503348

[B8] Scott S Cumberland P Shulman CE Cousens S Cohen BJ Brown DW Bulmer JN Dorman EK Kawuondo K Marsh K Neonatal measles immunity in rural Kenya: the influence of HIV and placental malaria infections on placental transfer of antibodies and levels of antibody in maternal and cord serum samples J Infect Dis 2005 191 11 1854 1860 10.1086/429963 15871118

[B9] Scott S Moss WJ Cousens S Beeler JA Audet SA Mugala N Quinn TC Griffin DE Cutts FT The influence of HIV-1 exposure and infection on levels of passively acquired antibodies to measles virus in Zambian infants Clin Infect Dis 2007 45 11 1417 1424 10.1086/522989 17990222

[B10] Communicable diseases surveillance Commun Dis Intell 1997 21 8 107 115 9140103 10.33321/cdi.1997.21.24

[B11] Kumar ML Johnson CE Chui LW Whitwell JK Staehle B Nalin D Immune response to measles vaccine in 6-month-old infants of measles seronegative mothers Vaccine 1998 16 20 2047 2051 10.1016/S0264-410X(98)00083-8 9796063

[B12] Cutts FT Grabowsky M Markowitz LE The effect of dose and strain of live attenuated measles vaccines on serological responses in young infants Biologicals 1995 23 1 95 106 10.1016/1045-1056(95)90018-7 7619443

[B13] Garly ML Bale C Martins CL Monteiro M George E Kidd M Dias F Aaby P Whittle HC Measles antibody responses after early two dose trials in Guinea-Bissau with Edmonston-Zagreb and Schwarz standard-titre measles vaccine: better antibody increase from booster dose of the Edmonston-Zagreb vaccine Vaccine 2001 19 15–16 1951 1959 11228365 10.1016/s0264-410x(00)00431-x

[B14] Labeaud AD Malhotra I King MJ King CL King CH Do antenatal parasite infections devalue childhood vaccination? PLoS Negl Trop Dis 2009 3 5 e442 10.1371/journal.pntd.0000442 19478847 PMC2682196

[B15] van Riet E Hartgers FC Yazdanbakhsh M Chronic helminth infections induce immunomodulation: consequences and mechanisms Immunobiology 2007 212 6 475 490 10.1016/j.imbio.2007.03.009 17544832

[B16] Malhotra I Mungai P Wamachi A Kioko J Ouma JH Kazura JW King CL Helminth- and Bacillus Calmette-Guerin-induced immunity in children sensitized in utero to filariasis and schistosomiasis J Immunol 1999 162 11 6843 6848 10352306

[B17] Malhotra I Mungai PL Wamachi AN Tisch D Kioko JM Ouma JH Muchiri E Kazura JW King CL Prenatal T cell immunity to Wuchereria bancrofti and its effect on filarial immunity and infection susceptibility during childhood J Infect Dis 2006 193 7 1005 1013 10.1086/500472 16518763

[B18] Webb EL Mawa PA Ndibazza J Kizito D Namatovu A Kyosiimire-Lugemwa J Nanteza B Nampijja M Muhangi L Woodburn PW Effect of single-dose anthelmintic treatment during pregnancy on an infant’s response to immunisation and on susceptibility to infectious diseases in infancy: a randomised, double-blind, placebo-controlled trial Lancet 2011 377 9759 52 62 10.1016/S0140-6736(10)61457-2 21176950 PMC3018567

[B19] Elliott AM Kizza M Quigley MA Ndibazza J Nampijja M Muhangi L Morison L Namujju PB Muwanga M Kabatereine N The impact of helminths on the response to immunization and on the incidence of infection and disease in childhood in Uganda: design of a randomized, double-blind, placebo-controlled, factorial trial of deworming interventions delivered in pregnancy and early childhood [ISRCTN32849447] Clin Trials 2007 4 1 42 57 10.1177/1740774506075248 17327245 PMC2643383

[B20] WHO Manual for the laboratory diagnosis of measles and rubella virus infection 2007 Second CH-1211 Geneva 27, Switzerland: the WHO Document Production Services, Geneva, Switzerland

[B21] Friend J Mackie & McCartney Practical Medical Microbiology 1996 Edinburgh: Churchhill Livingstone

[B22] Katz N Chaves A Pellegrino N A simple device for quantitative stool thick-smear technique in Schistomiasis Mansoni Rev Inst Med Trop Sao Paulo 1972 14 397 400 4675644

[B23] Bukusuba JW Hughes P Kizza M Muhangi L Muwanga M Whitworth JA Elliott AM Screening for intestinal helminth infection in a semi-urban cohort of pregnant women in Uganda Trop Doct 2004 34 1 27 28 14959970 10.1177/004947550403400113

[B24] Hillier SD Booth M Muhangi L Nkurunziza P Khihembo M Kakande M Sewankambo M Kizindo R Kizza M Muwanga M Plasmodium falciparum and helminth coinfection in a semi urban population of pregnant women in Uganda J Infect Dis 2008 198 6 920 927 10.1086/591183 18721060 PMC2886962

[B25] Muhangi L Woodburn P Omara M Omoding N Kizito D Mpairwe H Nabulime J Ameke C Morison LA Elliott AM Associations between mild-to-moderate anaemia in pregnancy and helminth, malaria and HIV infection in Entebbe, Uganda Trans R Soc Trop Med Hyg 2007 101 9 899 907 10.1016/j.trstmh.2007.03.017 17555783 PMC1950430

[B26] Woodburn PW Muhangi L Hillier S Ndibazza J Namujju PB Kizza M Ameke C Omoding NE Booth M Elliott AM **Risk Factors for Helminth, Malaria, and HIV Infection in Pregnancy in Entebbe,** Uganda PLoS Negl Trop Dis 2009 3 6 e473 10.1371/journal.pntd.0000473 19564904 PMC2696595

[B27] Melrose WD Turner PF Pisters P Turner B An improved Knott’s concentration test for the detection of microfilariae Trans R Soc Trop Med Hyg 2000 94 176 10.1016/S0035-9203(00)90266-9 10897361

[B28] Webb EL Kyosiimire-Lugemwa J Kizito D Nkurunziza P Lule S Muhangi L Muwanga M Kaleebu P Elliott AM The effect of anthelmintic treatment during pregnancy on HIV plasma viral load: results from a randomized, double-blind, placebo-controlled trial in Uganda J Acquir Immune Defic Syndr 2012 60 3 307 313 10.1097/QAI.0b013e3182511e42 22728750 PMC3383620

[B29] Date AA Kyaw MH Rue AM Klahn J Obrecht L Krohn T Rowland J Rubin S Safranek TJ Bellini WJ Long-term persistence of mumps antibody after receipt of 2 measles-mumps-rubella (MMR) vaccinations and antibody response after a third MMR vaccination among a university population J Infect Dis 2008 197 12 1662 1668 10.1086/588197 18419346 PMC9169514

[B30] Dietz V Rota J Izurieta H Carrasco P Bellini W The laboratory confirmation of suspected measles cases in settings of low measles transmission: conclusions from the experience in the Americas Bull World Health Organ 2004 82 852 857 15640921 PMC2623064

[B31] Spiegel A Tall A Raphenon G Trape JF Druilhe P Increased frequency of malaria attacks in subjects co-infected by intestinal worms and Plasmodium falciparum malaria Trans R Soc Trop Med Hyg 2003 97 2 198 199 10.1016/S0035-9203(03)90117-9 14584377

[B32] Urban BC Todryk S Malaria pigment paralyzes dendritic cells J Biol 2006 5 2 4 10.1186/jbiol37 16620370 PMC1561485

[B33] Luxemburger C McGready R Kham A Morison L Cho T Chongsuphajaisiddhi T White NJ Nosten F Effects of malaria during pregnancy on infant mortality in an area of low malaria transmission Am J Epidemiol 2001 154 5 459 465 10.1093/aje/154.5.459 11532788

[B34] Smedman L Silva MC Gunnlaugsson G Norrby E Zetterstrom R Augmented antibody response to live attenuated measles vaccine in children with Plasmodium falciparum parasitaemia Ann Trop Paediatr 1986 6 2 149 153 2425725 10.1080/02724936.1986.11748428

[B35] Helfand RF Witte D Fowlkes A Garcia P Yang C Fudzulani R Walls L Bae S Strebel P Broadhead R Evaluation of the immune response to a 2-dose measles vaccination schedule administered at 6 and 9 months of age to HIV-infected and HIV-uninfected children in Malawi J Infect Dis 2008 198 10 1457 1465 10.1086/592756 18828743

[B36] Farquhar C Wamalwa D Selig S John-Stewart G Mabuka J Majiwa M Sutton W Haigwood N Wariua G Lohman-Payne B Immune responses to measles and tetanus vaccines among Kenyan human immunodeficiency virus type 1 (HIV-1)-infected children pre- and post-highly active antiretroviral therapy and revaccination Pediatr Infect Dis J 2009 28 4 295 299 10.1097/INF.0b013e3181903ed3 19258919 PMC2779204

[B37] Abramczuk BM Mazzola TN Moreno YM Zorzeto TQ Quintilio W Wolf PS Blotta MH Morcillo AM da Silva MT Dos Santos Vilela MM Impaired humoral response to vaccines among HIV-exposed uninfected infants Clinical and vaccine immunology: CVI 2011 18 9 1406 1409 10.1128/CVI.05065-11 21775515 PMC3165213

[B38] Elliott AM Mawa PA Webb EL Nampijja M Lyadda N Bukusuba J Kizza M Namujju PB Nabulime J Ndibazza J Effects of maternal and infant co-infections, and of maternal immunisation, on the infant response to BCG and tetanus immunisation Vaccine 2010 29 2 247 255 10.1016/j.vaccine.2010.10.047 21040693 PMC3021124

[B39] McMurray DN Loomis SA Casazza LJ Rey H Influence of moderate malnutrition on morbidity and antibody response following vaccination with live, attenuated measles virus vaccine Bull Pan Am Health Organ 1979 13 1 52 57 427297

[B40] Neumann PWWJM Jessamine AG O’Shaughnessy MV Comparison of measles antihemolysin test, enzyme linked Immunosorbent assay, and hemagglutination Inhibition test with neutralisation test for determination of Immune status J Clin Microbiol 1985 22 2 3 10.1128/jcm.22.2.296-298.1985 PMC268379 3897272

[B41] Mbabazi WB Nanyunja M Makumbi I Braka F Baliraine FN Kisakye A Bwogi J Mugyenyi P Kabwongera E Lewis RF Achieving measles control: lessons from the 2002–06 measles control strategy for Uganda Health Policy Plan 2009 Oxford: Oxford University Press 10.1093/heapol/czp008 19282484

[B42] Baliraine FN Bwogi J Bukenya H Seguya R Kabaliisa T Kisakye A Mbabazi WB Smit SB Possible interruption of measles virus transmission, Uganda, 2006–2009 Emerg Infect Dis 2011 17 1 110 113 10.3201/eid1701.100753 21192868 PMC3204633

